# Rampant Increase in Cases of Mucormycosis in India and Pakistan: A Serious Cause for Concern during the Ongoing COVID-19 Pandemic

**DOI:** 10.4269/ajtmh.21-0608

**Published:** 2021-08-30

**Authors:** Behram Khan Ghazi, Sudhan Rackimuthu, Um Ul Wara, Anmol Mohan, Uzzam Ahmed Khawaja, Shkaib Ahmad, Shahzaib Ahmad, Mohammad Mehedi Hasan, Ana Carla dos Santos Costa, Shoaib Ahmad, Mohammad Yasir Essar

**Affiliations:** ^1^ Punjab Medical College, Faisalabad, Pakistan;; ^2^ Father Muller Medical College, Mangalore, Karnataka, India;; ^3^ Karachi Medical & Dental College, Karachi, Pakistan;; ^4^ Jinnah Medical and Dental College, Karachi, Pakistan;; ^5^ Dera Ghazi Khan Medical College, Punjab, Pakistan;; ^6^ Fatima Memorial Hospital College of Medicine & Dentistry, Lahore, Pakistan;; ^7^ Department of Biochemistry and Molecular Biology, Faculty of Life Science, Mawlana Bhashani Science and Technology University, Tangail, Bangladesh;; ^8^ Faculty of Medicine, Federal University of Bahia, Salvador, Bahia, Brazil;; ^9^ Department of Medicine and General Surgery, Punjab Medical College, Faisalabad, Pakistan;; ^10^ Kabul University of Medical Sciences, Kabul, Afghanistan

## Abstract

COVID-19 is a global health crisis that continues to pose new challenges all around the world. Amidst the growing pandemic, a spike in the number of mucormycosis cases in India and Pakistan has been reported in COVID-19 patients and in those who have recovered from COVID-19. This increase in cases may be related to the overuse of steroids and zinc, the use of industrial oxygen, unsterilized medical equipment, frequent intubation, a weakened immune system, and pre-existing comorbidities such as diabetes. As a result, it is critical to take steps to handle the current increase in mucormycosis cases. Therefore, this article aims to highlight the existence of mucormycosis amidst the COVID-19 pandemic in India and Pakistan, focusing on possible causes and implications, and suggests important plans of action to be taken during this pandemic.

## INTRODUCTION

The COVID-19 outbreak caused by the severe acute respiratory syndrome coronavirus 2 (SARS-CoV-2) was declared a global pandemic on March 11, 2020, by the WHO.^1^ As of May 28, 2021, a combined total of 28,459,007 confirmed cases, with 339,361 casualties in India and Pakistan, were reported due to COVID-19.^2^

In addition to the overlapping of symptoms with other infectious diseases, making the differential diagnosis difficult,^3^^–^^6^ the ongoing pandemic and its evolving mechanism of action have baffled physicians around the globe. According to a recent estimate for the year 2019–2020, the prevalence of mucormycosis ranged from 0.005 to 1.7 per million people worldwide, with the incidence in India about 80 times greater than in industrialized nations.^7^^,^^8^ At the beginning of May 2020, doctors across India became curious about a rise in mucormycosis cases. The majority of the infected were COVID-19 patients, recently recovered ones, immunosuppressed individuals, or individuals with underlying conditions, particularly diabetes.^9^^–^^11^ At least 14,872 cases of mucormycosis have been reported in India as of May 28, 2021.^12^ Two states have declared an epidemic, and the central government has declared it a prominent disease in India.^13^

In comparison, the situation in Pakistan is not as alarming as in India. Still, it is believed that the cases might continue to increase if appropriate precautions are not taken.^14^ Even though there exists a lack of data on the actual disease burden of mucormycosis in Pakistan, a recent study shows increasing trends of mucormycosis in developing countries.^15^

The black fungus occurs due to mold (mucormycetes) present in damp environments like soil or compost and can invade the respiratory tract. It does not spread via person-to-person contacts and is not contagious. As per the CDC, these fungi are not harmful but can seriously affect people with weakened immune systems. Black fungus targets the sinuses or lungs after fungal spores are inhaled from the air and might also affect skin after a surface injury like a cut or a burn. Symptoms predominantly depend on where the fungus is growing or infiltrated in the body and can also spread to the eyes and the brain, leading to catastrophic outcomes. According to the CDC, a study conducted in 2005 and involving 929 cases dating back to 1885 found an overall mortality rate of 54%. The determinants of the mortality rate are the type of fungus involved and part of the body affected.^13^

Susceptibility to infection increases in immunocompromised individuals, including COVID-19 patients, diabetic patients, people on steroids, and individuals with comorbidities like cancer or organ transplants.^10^ COVID-19 patients are susceptible specifically not only because the virus affects their immune system but because the therapeutic regimen used for severe cases (e.g., steroids) can also suppress their immune response. COVID-19 patients admitted to intensive care units receiving oxygen therapy might encounter humidifiers in the ward, increasing their exposure to moisture and thus making them more susceptible to fungal infection. As per data from the International Diabetes Federation, the prevalence of diabetes in adults in India is 8.9%, with about 77 million patients suffering from diabetes.^16^ Even more worrisome is the proportion of the population with diabetes in Pakistan, which is higher than 10%.^17^ A study from Pakistan also highlighted the increased number of fungal infections in patients with severe COVID-19 disease.^18^

## POTENTIAL CAUSES FOR THE INCREASE IN CASES OF MUCORMYCOSIS

Steroids are a part of the regimen used in treating patients with severe COVID-19 disease.^19^ This drug class acts by decreasing the patient’s immune response, making the patient susceptible to secondary infections. The prolonged use of steroids is considered one of the possible reasons for the emergence of mucormycosis or black fungus. Steroids are also used to decrease the patient’s sugar levels and raise the blood glucose level by causing insulin resistance, thus reducing insulin action.^20^ Further, many patients are resorting to self-medicating at home without proper knowledge as a result of the collapsing healthcare system due to the ongoing COVID-19 pandemic. This has been a recurring concern, especially in India and Pakistan.^21^^,^^22^ Another possible contributing factor could be the constant but incomprehensive media coverage over the use of steroids to treat COVID-19, possibly resulting in inadequate knowledge gained by the general public to self-medicate with steroids even when not deemed necessary.

In addition, according to some experts, black fungus has a preference for manifesting in individuals with uncontrolled diabetes. With India being deemed as the diabetes capital of the world, augmented with noncompliance to medication by many, the control of diabetes in most Indian patients is currently quite poor.^23^ Experts have also speculated that the climate of South Asia can also be a contributing factor because the growth of fungus has been associated with high temperature and humidity.^24^ The concurrent rise in cases of mucormycosis in India and Pakistan could therefore be possibly attributed to similarities in their climate.^25^ Contaminated water used in humidifiers, the use of industrial oxygen, unsterilized medical equipment, and prolonged use of the same masks and tubes is also strongly believed to cause mucormycosis.^26^ An unhygienic environment, poverty, and lack of living standards, which have been further worsened due to lockdowns to contain the spread of COVID-19, may also have played a role in this fungal outbreak in India and Pakistan.^27^

Since the beginning of the pandemic, plenty of supplements, including vitamin C, vitamin D, and zinc, have managed to find their place as a part of the treatment regimen against COVID-19 to help boost immunity. However, zinc is known to play an important role in fungal growth and development and positively influences fungal pathogenesis. Zinc also possibly helps regulate the expression of many proteins required to cause infection.^28^ To halt fungal growth, mammalian hosts typically reduce the levels of free zinc and other metals. The inadvertent overuse of zinc during the COVID-19 pandemic has resulted in an overall increase in its levels in the body, which could also be a potential cause for the rise in mucormycosis cases.^9^

## IMPLICATIONS OF MUCORMYCOSIS DURING COVID-19

India and Pakistan, as developing countries, have been struggling with COVID-19 and its repercussions for almost a year and a half. Consequently, their healthcare systems have also been severely affected and brought to their knees,^29^^–^^31^ affecting the fight against other infectious diseases. In the middle of such crises, the advent of black fungus has put a great deal of strain on healthcare workers and the government. They are already exhausted mentally, physically, and financially.

The demand for the antifungal drug amphotericin B, the sole suggested and effective treatment of black fungus, has risen in recent weeks due to increasing mucormycosis cases. The surging demand for antifungal medication has created an acute shortage, giving rise to a black market for drugs that were already too expensive for most people to afford.^32^^,^^33^

With India’s continuous battle with COVID-19, hospitals are running out of beds, ventilators, and oxygen cylinders, which has already put a strain on the budget as well as state’s healthcare infrastructure; meanwhile, treating this condition is proving to be challenging because it requires multidisciplinary expertise. In an overwhelmed healthcare system, finding hospitals where mucormycosis patients can get surgery and postoperative care can be another logistical nightmare.

Eye, nose, and throat specialists expect to see more mucormycosis cases in the coming weeks as India continues to face a crippling second wave of COVID-19 saturating the healthcare system, which is now barely keeping afloat. An intensifying burden has been hastening the diminution in the available workforce and resources required for effective management.^30^ In addition, the surfacing of another outbreak, such as that of mucormycosis, will prove more disastrous because the implementation of steps to treat the outbreak effectively would be an arduous task due to the ramifications of ongoing COVID-19. Even though the number of cases of mucormycosis in Pakistan is comparatively lower than that of India, there are several pitfalls in the overall healthcare system that could augment the further rise in cases.^14^ Surveillance of communicable and noncommunicable diseases is limited in Pakistan due to the absence of a robust national healthcare system, leading to the inability to gauge the actual burden of a particular disease or infection. Moreover, the situation is further rendered problematic by the poor fungal diagnostic capabilities of most laboratories in Pakistan, poor infection control practices, inadequate antimicrobial stewardship, and the lack of essential antifungal drugs.^34^

## ACTION PLAN AND RECOMMENDATIONS

With the number of mucormycosis cases steadily rising both in India and Pakistan, it is crucial to take steps to help mitigate the outbreak. The Indian Council of Medical Research has recently released an advisory for the effective screening, diagnosis, and management of the disease during the ongoing COVID-19 pandemic.^35^ Predisposing conditions for mucormycosis include uncontrolled diabetes mellitus, long-term steroid use, prolonged intensive care unit stay, post-transplant patients, and individuals on voriconazole therapy.^36^ It is therefore imperative for these patients to exercise caution and be more vigilant by adhering strictly to public health guidelines and advice for COVID-19 as well as for mucormycosis. A few critical acts of prevention, such as 1) using masks when visiting construction sites; 2) wearing shoes, long trousers, long sleeve shirts, and gloves while handling soil, moss, or manure; and 3) maintaining personal hygiene by thorough scrubbing while taking a bath, are all believed to be at the forefront in effectively preventing the spread of mucormycosis infection.

Because mucormycosis has a high propensity to turn fatal if left uncared for, it is important to be aware and not overlook the signs and symptoms of the disease, especially in individuals with COVID-19 and in diabetic and immunosuppressed individuals, for timely diagnosis and treatment. Symptoms such as nasal blockage or congestion, nasal discharge (blackish or bloody), local pain over the cheekbone, unilateral facial pain, numbness or swelling over the face, blackish discoloration over the bridge of nose or the palate, toothache, loosening of teeth, pain in the jaw, blurred or double vision, fever, skin lesion resembling that of eschar, chest pain, hemoptysis, and worsening of respiratory symptoms necessitate immediate concern, and one should try to report to the nearest health facility as soon as possible.^36^

Strict glucose level monitoring in people with diabetes and patients recovering from COVID-19 postdischarge is recommended to prevent mucormycosis infection. Maintaining a sterile and clean water supply and piping for humidifiers and vents can reduce the risk of mucormycosis. One must also not hesitate to request aggressive investigations, such as potassium hydroxide staining and microscopy, culture, and matrix-assisted laser desorption ionization time of flight mass spectrometry to discern a fungal etiology that may prove vital in clinching the diagnosis.^35^ Song et al.^37^ have advised a criterion for the prompt diagnosis and management of common invasive fungal infections (*Aspergillus*, candidiasis, cryptococcosis, and mucormycosis). Treatment of black fungus includes antifungals, mostly intravenously, as per CDC guidelines. The widely used medicines are amphotericin B, currently a first-line option in India for fighting the pandemic. Patients might require around 6 weeks of antifungals to recover. Surgery is often performed to remove dead or infected tissue.^38^

Furthermore, the uncontrolled use of drugs for treatment and self-medication being problematic, especially in countries like India and Pakistan. Therefore, it is empirical to address the issue by targeting policymakers, physicians, and the general public. Enforcement of strict laws to regulate the dispensing of drugs by pharmacists and other health establishments could help curtail the extent of self-medication by patients. An ordinance about the mandatory need for a prescription of a larger cohort of drugs, including steroids, antibiotics, and antifungals, for their procurement and use could help ease the problem at hand. A system to review, monitor, and regularly audit the treatment and drugs prescribed by a physician could help identify uncontrolled and unnecessary use of drugs, and lessons could be learned for more judicial use in the future. With further increases in mucormycosis cases across the countries, the health resources required for effective management also need to be mobilized, such as the drug amphotericin B, which is currently scarce in several states in India.^32^ Setting up a national database of relevant information of patients diagnosed with mucormycosis will most definitely also aid in monitoring and mapping of the disease across the country.

## CONCLUSION

COVID-19 is potentially linked to a high rate of secondary bacterial and fungal infections, most likely due to immune dysregulation. Furthermore, the uncontrolled use of steroids, monoclonal antibodies, and broad-spectrum antibiotics as part of the COVID-19 armamentarium can lead to fungal diseases or worsen pre-existing fungal diseases. Physicians should be aware of the possibility of invasive secondary fungal infections in patients with COVID-19 infection, especially in those who have pre-existing risk factors and comorbidities, and hence be able to detect and manage them early to help reduce mortality and morbidity. Steps must be taken to oversee and closely control the use of all therapeutic agents used in the treatment of COVID-19 patients to achieve the best possible therapeutic result while mitigating the likely consequence of acquiring mucormycosis. A call for multidisciplinary action involving all the stakeholders is therefore required to end this crisis.
